# Preventive effect of toothpastes with MMP inhibitors on human dentine erosion and abrasion *in vitro*

**DOI:** 10.1590/1678-775720150289

**Published:** 2016

**Authors:** Angelica Reis Hannas, Melissa Thiemi Kato, Cristiane de Almeida Baldini Cardoso, Ana Carolina Magalhães, José Carlos Pereira, Leo Tjäderhane, Marília Afonso Rabelo Buzalaf

**Affiliations:** 1 - Universidade de São Paulo, Faculdade de Odontologia de Bauru, Departamento de Ciências Biológicas, Bauru, SP, Brasil.; 2 - Universidade do Sagrado Coração, Departamento de Odontologia, Bauru, SP, Brasil.; 3 - Universidade do Sagrado Coração, Departamento de Odontopediatria, Bauru, SP, Brasil.; 4 - Universidade de São Paulo, Faculdade de Odontologia de Bauru, Departamento de Dentística, Endodontia e Materiais Odontológicos, Bauru, SP, Brasil.; 5 - Oulu University Hospital and University of Oulu, Medical Research Center, Institute of Dentistry, Oulu, Finland.

**Keywords:** Erosion, Dentin, Matrix metalloproteinases, Inhibitors, Toothpastes

## Abstract

The use of gels and mouthrinses with MMP inhibitors (chlorhexidine, and green tea extract) was shown to prevent erosive wear. The aim of this study was to analyze the protective effect of toothpastes containing MMP inhibitors on dentine loss induced by erosion *in vitro*.

**Material and Methods:**

Five groups each containing 12 specimens of human root dentine were prepared. The specimens were subjected to 1 min erosion by immersion in a cola drink, 4 times a day, for 5 d. Each day, after the first and last erosive challenges, the specimens were brushed for 15 s with a slurry of dentifrice and water (1:3) containing placebo, 1,100 ppm fluoride, 0.61% green tea extract, 0.12% chlorhexidine or 0.004% chlorhexidine (commercial toothpaste). Between the acid challenges, the specimens were stored in artificial saliva with remineralizing potential until the next treatment. Dentine loss was determined using profilometry. Data were analyzed using one-way ANOVA after log transform (p<0.05).

**Results:**

The mean wear values (μm) were as follows: placebo 1.83±0.53; 0.61% green tea extract 1.00±0.21; fluoride 1.27±0.43; 0.12% chlorhexidine 1.19±0.30; and 0.004% chlorhexidine 1.22±0.46. There was a significant difference in wear between placebo and all the treatment toothpastes, which did not differ from each other.

**Conclusion:**

The results suggest that toothpastes containing MMP inhibitors are as effective as those based on NaF in preventing dentine erosion and abrasion.

## INTRODUCTION

The reduction in caries prevalence, associated with an increase in tooth-retention rates, has posed new challenges regarding preventive measures in oral health. The augmented consumption of acidic foods and beverages has led to higher occurrence of dental erosion, which is defined as the loss of tooth substance caused by acids (pH<4.5), undersaturated regarding apatite, in the absence of microorganisms^15^ ^,^ ^22^. The frequent ingestion of acidic beverages has a profound effect on dentine, especially for adults and for the elderly population. With age, gingival recession is a common occurrence, leading to the subsequent exposure of cement and root dentine that are quite vulnerable to erosion^2^ ^,^ ^13^.

The exact mechanism of the erosion process in exposed dentine root surfaces is not known. Although the mineral phase of the dentine is lost after the erosive challenge, the fate of the organic phase is not completely understood^2^ ^,^ ^11^ ^,^ ^29^. When dentine is exposed to acids, the minerals from the peritubular/intertubular dentine junction are initially extracted. Next, the peritubular dentine is degraded and the dentine tubuli become wider^25^. Finally, a superficial layer of demineralized collagenous matrix can be detected. This demineralized organic layer is resistant to mechanic removal by brushing forces up to 4 N^5^ ^,^ ^8^, and it is plausible that this outermost layer of collagenous matrix might protect the remaining dentine against mechanical forces, like toothbrush abrasion^8^. It could also limit ionic diffusion into and out of the demineralized surface^16^ ^,^ ^19^ ^,^ ^21^. Together, these properties may explain why its maintenance has been attributed to reduce the progression of dentine erosion^2^ ^,^ ^3^.

It is not known to which extent the protective effect of the demineralized organic matrix is relevant in clinical reality. This layer could be degraded by matrix metalloproteinases (MMPs)^2^ ^,^ ^10^ ^,^ ^28^, which have been suggested to contribute to the progression of dentine erosion^2^ ^,^ ^11^. MMP inhibitors, such as chlorhexidine, green tea polyphenols, and FeSO_4,_ in solutions or gels, reduce the dentine erosive wear^4^ ^,^ ^17^ ^,^ ^18^ ^,^ ^24^. However, the use of toothpastes as a vehicle to deliver MMPs to dentine has not been studied so far. This vehicle seems particularly attractive, since toothbrushing with toothpaste is an integral part of daily oral hygiene measures, and the demineralized organic matrix is not removed during this procedure^5^ ^,^ ^8^. Thus, the present study tested the null hypothesis that toothpastes containing MMP inhibitors do not exert any influence on dentine erosion associated with toothbrush abrasion.

## MATERIAL AND METHODS

### Preparation of dentine specimens

**Figure 1:**
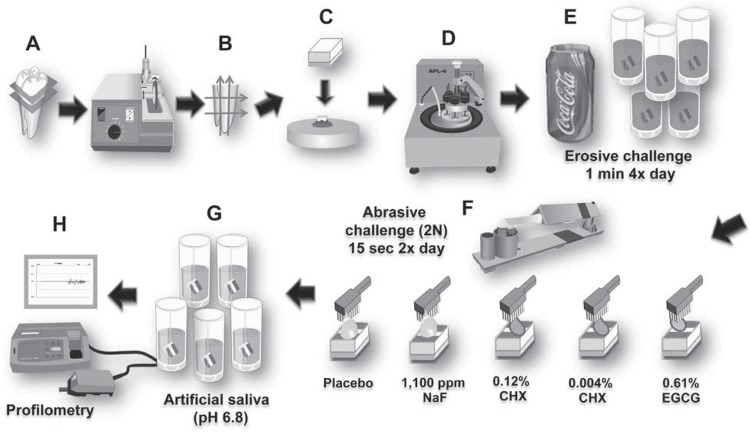
Schematic illustration of the experimental sequence. (A) The crowns were sectioned from the roots with a diamond saw. (B) Dentine slabs (4x4 mm) were cut from the cervical third of the root human root, (C) fixed on acrylic rods with sticky wax, and (D) the external surfaces were ground flat and polished with water-cooled carborundum discs (320, 600, and 1,200 grades of Al2O3 papers). Dentine specimens (n=12) were randomly divided into 5 groups. (E) The specimens were subjected to erosion (1 minute) by immersion in a cola drink, 4 times a day, for 5 d. (F) After the first and last erosive challenges, the specimens were brushed for 15 s with slurry of dentifrice and water (1:3) with the following treatment toothpastes: placebo; 1,100 ppm F as NaF; 0.61% green tea extract (EGCG); 0.12% chlorhexidine (CHX); 0.004% chlorhexidine (CHX). (G) The specimens were then stored in artificial saliva until the next treatment. (H) Dentine alterations were determined using profilometry

Sixty recently extracted non-carious human third molars were used in this study. The protocol was approved by the Institutional Review Board of Bauru School of Dentistry, University of São Paulo (011/2008). Prior to the extraction, the patients were informed about the use of their teeth for research purposes and consent was obtained. Teeth were stored in 0.9% NaCl with 0.2% NaN_3_ at 4°C. The crowns were sectioned from the roots with a diamond saw (Isomet 1000; Buehler, Lake Bluff, IL, USA). Dentine slabs (4x4 mm) were cut from the cervical third of the root under water cooling, mounted on acrylic blocks and ground flat with water-cooled carborundum discs (320, 600 and 1,200 grades of Al_2_O_3_ papers; Buehler). After each disc, the specimens were cleaned in ultrasound bath (T7 Thornton, Unique Ind. e Com. Ltda., São Paulo, SP, Brazil) for 2 min. Finally, the specimens were polished with felt paper wet by diamond spray (1 µm; Buehler) on a rotating polishing machine. The specimens were randomly assigned into five groups of 12 specimens each that differed according to the type of toothpaste used. Sample size was calculated based on a previous study^26^. In order to establish reference surfaces for lesion depth determination, two layers of nail varnish (Risqué: Niasi Indústria de Cosméticos LTDA, Taboão da Serra, SP, Brazil) were applied on each side of the surface of each specimen, leaving 1 mm of the middle unprotected (Figure 1).

### Treatment protocol

For erosive demineralization, each specimen was immersed in 30 mL of Coke^®^ (Cia de Bebidas Ipiranga, Ribeirão Preto, SP - Brazil, pH 2.6) for 1 min, 4 times a day, during 5 d. Between the demineralization phases, the samples were stored in artificial saliva^20^ containing 0.2 mM glucose, 9.9 mM NaCl, 1.5 mM CaCl_2_.2H_2_O, 3 mM NH_4_Cl, 17 mM KCl, 2 mM NaSCN, 2.4 mM K_2_HPO_4_, 3.3 mM urea, 2.4 mM NaH_2_PO_4_, and traces of ascorbic acid (pH 6.8) at 37°C for 59 min to allow remineralization. Each day, following the first and last erosive challenges, brushing was performed for 15 s *per* specimen using powered toothbrush with 166 oscillations/s (Oral-B^®^, Cross Action Power, Procter & Gamble, Cuajimalpa, Mexico). For standardized brushing force, a custom-made device was used, allowing for a pressure of approximately 2 N^32^. The brushing heads were renewed after 2.5 days of treatment. For each specimen, brushing was performed with 1 mL of a 1:3 (w/v) slurry of toothpaste and deionized water (Figure 1). Placebo toothpaste gel was prepared with the same composition and pH (7.0) of the other experimental toothpastes and with no active compound. Its composition included ethanol, water, sorbitol, glycerine, carboxymethylcellulose, xanthan gum, poliethyleneglycol 400, methylparaben, propylparaben, silicon dioxide, abrasive silicon dioxide, sodium lauryl sulphate, cocoamidopropylbetain, sodium saccharine, mint essence, and orthophosphoric acid. This also served as the base for the experimental gels. Three experimental toothpastes with a similar composition and pH (7.0) were prepared, with the exception of the compound tested, as follows: 1,100 ppm fluoride (as NaF, Sigma, Steinheim, Germany), 0.61% green tea extract (OM24, 100% *Camellia sinensis*-leaf extract with 30±3% catechin; Omnimedica, Schlieren, Switzerland), 0.12% chlorhexidine digluconate (Sigma). Commercial toothpaste containing 0.004% chlorhexidine digluconate (Elgydium, Pierre Fabre, Switzerland, pH 7.0) was also tested (Figure 1). After completion of the 5-day brushing, the specimens were rinsed with deionized water and stored in artificial saliva until analyzed (Figure 1).

### Dentine loss assessment

The nail varnish over the surfaces was carefully removed with a scalpel. The specimens were maintained wet to avoid shrinkage. Dentine tissue loss (µm) was quantitatively determined by a contact profilometer (Mahr Perthometer, Mahr, Göttingen, Germany) as described before^3^. Briefly, the specimens were slightly dried, gently removing only the excess of water with filter paper, and immediately analyzed. The diamond stylus was moved from one reference area to another across the exposed area (2.5 mm length and 2.0 mm width), recording the profile with a computer program (Software Mahr Surf XT20, Mahr). Five profile measurements were performed in the center of each specimen at intervals of 0.5 mm. The vertical distance between the midpoints of regression lines on the reference and experimental areas was defined as tissue loss (µm). The accuracy of the method, evaluated in previous studies, is around 0.5 µm.

### Statistical analysis

After checking for equality of variances and normal distribution of errors, data were analyzed by one-way ANOVA and Tukey’s test after log transformation. The software GraphPad Instat for Windows version 3.0 (La Jolla, CA, USA) was used. The significance level was set at 5%.

## RESULTS

**Figure 2:**
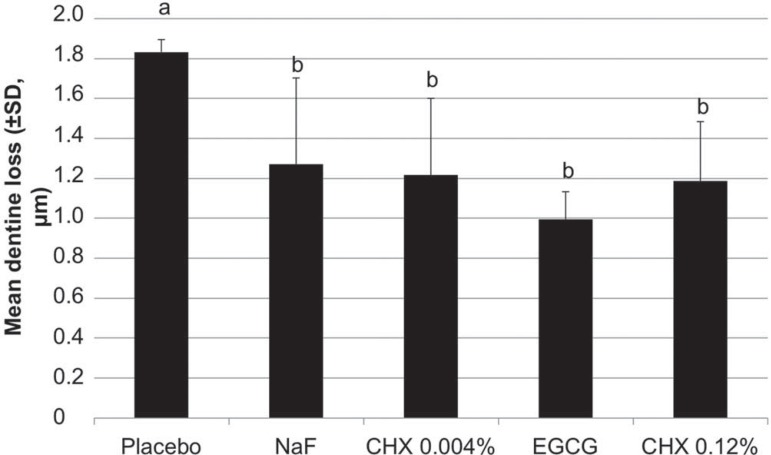
Mean loss of dentine (± standard deviation) in groups submitted to erosive and abrasive challenges *in vitro*, treated with toothpastes containing MMP inhibitors or fluoride. Distinct letters indicate significant difference among the toothpastes (ANOVA after log transform, p<0.05)

The mean (±SD) dentine loss (μm) found for the specimens treated with the placebo toothpaste (1.83±0.53) was significantly higher (F=5.353, p=0.008) than the values found for the specimens from the toothpastes containing MMP inhibitors (1.00±0.21, 1.19±0.30, and 1.22±0.46 for 0.61% green tea extract, 0.12% chlorhexidine or 0.004% chlorhexidine, respectively) or fluoride (1.27±0.43). The toothpastes containing active compounds, however, were not significantly different from one another (Figure 2).

## DISCUSSION

The study design aimed to simulate the habits of patients with erosive lesions. The total dentine erosion time was 20 min with storage in remineralizing solution between the acidic challenges to allow clinically relevant remineralization. The total abrasion time throughout the study was 2.5 min, which would correspond to 30 brushings of 5 s each for one tooth in the clinical condition^31^. Despite the experimental *in vitro* conditions employed in an attempt to resemble as closely as possible the clinical condition, some situations could not be reproduced by our protocol, such as the presence of the acquired pellicle, the dynamic salivary flow, the presence of bacteria, as well as variations in temperature. In addition, the concentration of the actives used in the experimental dentifrices (0.61% green tea extract and 0.12% chlorhexidine digluconate) had a good effect on the reduction in dentine erosion when added to rinse solutions in a previous *in situ* study^24^.

The rate of loss in dentine reduces along time if the demineralized organic matrix is not removed^3^ ^,^ ^6^. The demineralized collagen layer works as a diffusion obstacle, which occurs also in root caries^27^. It has also been speculated that this organic layer may present buffering capacity. During an erosive challenge from the outer surface, it may adsorb H^+^, therefore protecting the inner dentine from pH decrease^2^ ^,^ ^19^. The presence and maintenance of the organic dentine matrix, through the inhibition of collagenolytic enzymes present in dentine and saliva, is required for the remineralization of eroded dentine^12^ ^,^ ^30^. As far as we know, this is the first study to assess the effect of toothpastes with MMP inhibitors on dentine loss and the second *in vitro* study to show that dentinal enzymes have a role in erosion progression^1^. Most of the previous studies have been performed *in situ,* in which the potential role of salivary MMPs cannot be differentiated from the dentinal MMPs. Since demineralized dentine organic matrix seems to be quite resistant to mechanical forces, even under high brushing forces^7^, avoiding its degradation by employing MMP inhibitors has emerged as a promising method to prevent dentine erosion. This approach has been tested by using rinses and gels as vehicles to deliver MMP inhibitors^17^ ^,^ ^18^ ^,^ ^24^. However, even though fluoride toothpastes are widely tested to prevent dental erosion^23^, to our knowledge, they have never been used as vehicles to deliver MMP inhibitors to the dental structure.

The results of the present investigation demonstrated that toothpastes with chlorhexidine or green tea extract are able to decrease dentine loss under mild *in vitro* erosive and abrasive conditions. Additionally, they were at least as effective as conventional toothpaste with 1,100 ppm F. Both chlorhexidine and green tea catechins are MMP inhibitors^4^ ^,^ ^9^ ^,^ ^24^ with unknown remineralizing potential. As a matter of fact, while both hesperidin (a citrus fluid flavonoid) and chlorhexidine reduced the collagenase-induced loss of mineral and erosive lesion progression, only hesperidin was able to induce mineral uptake in a recent study^14^. The present investigation supports previous studies with reduced dentine erosion using different MMP inhibitors with distinct vehicles^17^ ^,^ ^18^ ^,^ ^24^, and further indicates the distinctive role of MMPs in the progression of dentine erosion. Thus, the null hypothesis was rejected.

In the present study, the toothpastes tested contained MMP inhibitors or fluoride. However, since the effect of fluoride to prevent dentine erosion has been suggested to be dependent on the maintenance of the demineralized organic matrix^2^ ^,^ ^7^ ^,^ ^16^ ^,^ ^30^, it would be interesting to test toothpastes containing both MMP inhibitor(s) and fluoride. It is important to highlight that the efficacy of MMP inhibitors to prevent dentine erosion seems to be dependent on the vehicle used to deliver these inhibitors. The single application of gels^17^ is more effective than multiple rinses^24^ under similar erosive protocols. In fact, this application of gels containing EGCG or chlorhexidine completely prevented dentine erosion under erosive challenges conducted *in situ*/*ex vivo* (100 min of erosion)^17^. However, when the erosive challenges were conducted for 10 d (200 min of erosion), some degree of dentine loss occurred (Kato, et al., unpublished observations). Thus it seems that even in the best case scenario, repeated MMP inhibitor applications are needed for permanent prevention. This would be easier to achieve with the daily oral hygiene products, especially toothpastes, with MMP-inhibiting action.

## CONCLUSION

In conclusion, under the limitations of this *in vitro* study, toothpastes containing MMP inhibitors (0.12% or 0.004% chlorhexidine, or 0.61 % green tea extract) were able to prevent dentine wear caused by erosion and abrasion. They performed similarly to the toothpaste containing 1,100 ppm fluoride, which was able to partially reduce dental erosion. Since MMP inhibition has also been suggested as a strategy to reduce dentine caries progression^28^ ^,^ ^30^, toothpaste with MMP inhibiting action combined with fluoride might be effective in the protection of the two most common reasons for the loss of tooth structure, caries and erosion. However, these results should be confirmed in further *in situ* and clinical studies.
